# Non-allergic factors that influence asthma control in pregnancy

**DOI:** 10.18332/ejm/191295

**Published:** 2024-08-28

**Authors:** Agnieszka Rey, Marta Chełmińska, Iwona Damps-Konstańska

**Affiliations:** 1 Department of Pulmonology and Allergology, Medical University of Gdańsk, Gdańsk, Poland

**Keywords:** pregnancy, asthma, perinatal outcomes

## Abstract

**INTRODUCTION:**

Numerous factors may influence the asthma course during pregnancy, potentially elevating the risk of specific pregnancy complications. This study aimed to evaluate non-allergic factors influencing asthma and to assess perinatal outcomes between asthmatic and non-asthmatic pregnancies in the population of the Pomeranian Voivodeship region of Poland.

**METHODS:**

The mixed cohort study was performed with 83 pregnant asthmatic patients aged 18–38 years. The control group consisted of 83 patients without asthma diagnosis or symptoms. A specially designed questionnaire was used to evaluate asthma course and perinatal outcomes. An Asthma Control Test (ACT) adapted for pregnancy was performed on enrollment. Asthma severity was assessed according to GINA guidelines.

**RESULTS:**

In 19 cases (22.80%), patients quit their regular treatment after pregnancy was confirmed. Respiratory tract infection occurred in 23 patients (27.71%) and had been statistically significantly more frequent among patients with partially and uncontrolled asthma (χ^2^=8.504, p<0.05). No statistically significant difference was found between infection episodes and perinatal complications. The incidence of cesarean section was significantly higher among patients with asthma (χ^2^=16.37, p<0.01), particularly in patients with severe asthma (χ^2^=7.07, p<0.05) and uncontrolled asthma (χ^2^=6.7, p<0.05). Apgar score was statistically significantly lower in patients with severe asthma (χ^2^=20.37, p<0.05).

**CONCLUSIONS:**

Respiratory tract infections and adequate asthma treatment are the most important modifiable factors in preventing perinatal complications associated with asthma.

## INTRODUCTION

Asthma is one of the most common chronic diseases complicating pregnancy, affecting up to 12% of patients^1^. It has been linked with a range of pregnancy-related complications. Maternal complications include gestational diabetes^2,3^, perinatal hemorrhage^2,4^, placental defects^2^, pre-eclampsia^3,5^, and cesarean section^6,7^. Fetal complications encompass congenital defects (cleft palate, gastroschisis)^8,9^, preterm birth^1,10^, low birth weight^10^, longer hospitalization time of a newborn, and even newborn death^9,11^.

Numerous factors may contribute to the pregnancy complications observed in asthmatic patients, with exacerbations and severe or uncontrolled asthma identified as particularly significant^12^. Factors influencing exacerbations and contributing to worse asthma control may be divided into modifiable and non-modifiable. Among modifiable factors, the discontinuation of treatment is of utmost significance. Although principles of treatment of asthma in pregnancy are adequate to those concerning non-pregnant patients and asthma treatment is considered safe during pregnancy^1,13^, many studies show a tendency to reduce treatment in pregnant women, not only by patients themselves^14,15^, but also by medical personnel^5^. It is alarming, as proper treatment is considered to prevent most pregnancy complications linked with asthma^13,16^. Another modifiable factor is smoking. Complications of smoking in pregnancy are generally well recognized. In asthmatic patients, smoking is linked with an increased risk of asthma exacerbations, preterm birth, and urinary tract infections^17^. Despite that, the prevalence of smoking among pregnant patients with asthma is relatively high (up to 34%)^1^. Respiratory tract infection may be classified within modifiable factors. Due to immunological changes influenced by pregnancy and present in asthma, viral infections last longer and present more intense symptoms in this group of patients^17^. Preventive procedures, including vaccinations, e.g. for influenza and COVID-19, are recommended to lower the risk of infection. Non-modifiable risk factors include multiple pregnancies, age over 35 years, black race, and concurrent disorders like obesity, depression, and anxiety^18^. It is suggested that patients with the presence of non-modifiable factors may need more thorough monitoring^12^.

Maternal asthma may influence prenatal outcomes and subsequent stages of life. An association has been observed between uncontrolled asthma and the development of early childhood asthma in offspring^19^.

The effective management of pregnant patients with asthma is teamwork. Specialist care focuses mainly on proper treatment adjustment of severe or uncontrolled asthma. Midwives, as frontline healthcare providers, are playing a pivotal role in preventing adverse perinatal outcomes. In the context of asthma, this could involve identifying patients predisposed to asthma exacerbations, discerning their risk factors, or utilizing questionnaires assessing asthma control to pinpoint individuals susceptible to adverse perinatal outcomes associated with the disease. They could also promote medication adherence and advocate for expert consultation when there are concerns about suboptimal asthma treatment or the necessity for referral to other specialists.

The issue of asthma’s influence on pregnancy outcomes has been extensively explored in global literature. However, it is not sufficiently covered in studies based on the Polish population. Only studies concerning asthma in pregnancy were conducted in the last 20 years and covered only FENO (fractional concentration of exhaled nitric oxide) use in asthma management^20,21^.

The study aimed to evaluate non-allergic, potentially avoidable factors that may influence pregnancy course in asthmatic patients and to compare perinatal outcomes between asthmatic and non-asthmatic pregnant women in the population of Pomeranian Voivodeship region in Poland.

## METHODS

### Study design

This cohort design study, which used both retrospective and prospective data collection, was performed on 83 asthmatic patients.

### Setting and participants

The study was performed in 2015–2020; patients were recruited in the Department of Allergology of MUG and the Allergology Outpatient Departments of Pomeranian Voivodeship. All women signed written informed consent for participation. Patients in a prospective arm were enrolled throughout pregnancy, predominantly in the second trimester. Patients in the retrospective group were enrolled up to 5 years after childbirth, predominantly in the six-week postpartum period, providing that there was complete medical documentation available. Questionnaires were introduced by trained personnel, informed about the aim of the study, questionnaire structure, and inclusion and exclusion criteria. A telephone interview with authors followed the completion of the questionnaire, and (in the case of the prospective group) a second telephone interview was performed 1–2 months after childbirth. Patients who did not respond to the first telephone interview during pregnancy were reassigned to the retrospective group. Data were supplemented according to available documentation, further standardized, and recorded in the database.

The inclusion criteria encompassed the following: age range 18–40 years, a diagnosis of asthma in accordance with GINA guidelines, and singleton pregnancy. Exclusion criteria were defined as the failure to meet the established inclusion criteria. Approval was provided by the Medical University of Gdansk’s Ethics Committee NKBN/415/2015.

### Measurements

In the study on enrollment and after delivery, we used a questionnaire developed by authors for the purpose of the study, with questions concerning perinatal outcomes, including mode of delivery, gestation age at partum, birth weight, Apgar score, presence of pregnancy and peripartum complications. A large section was dedicated to asthma courses during pregnancy, including asthma control, treatment, occurrence of exacerbations, and possible causes of insufficient asthma control. The study ended at the beginning of the SARS-CoV-2 pandemic hence information concerning COVID-19 was not included in the study.

The asthma diagnosis was confirmed in medical documentation according to the GINA guidelines valid upon enrollment. Apart from a questionnaire, an asthma control test (ACT) was performed during enrollment. ACT is a validated tool for assessing asthma control. It consists of five questions concerning asthma symptoms, the need for rescue treatment, and a maximum score of 25 points. A score of 25 points suggests good asthma control, below 20 poor asthma control, and 20–24 partially controlled asthma. The test was adjusted for pregnancy according to findings from previous studies (Cronbach’s alpha for internal consistency was similar across time points, 0.84–0.90)^22^. According to GINA guidelines that were valid on enrollment, asthma severity was assessed according to the step of current (past three months) medication^13^. Exacerbations of asthma were defined as a transient loss of asthma control with a need for additional asthma treatment and were divided into mild, moderate, and severe. Mild exacerbations were defined as a need for reliever use, moderate as an increase of the treatment step, and severe as a need for hospitalization following symptoms aggravation. Asthma worsening was defined as the presence of perceived worsening asthma symptoms during pregnancy, as reported by the patient. The Apgar score was used to evaluate the neonatal status of neonates born on a term by summing numerical scores (0–2) for activity (muscle tone), pulse (heart rate), grimace (reflex irritability), appearance (skin color), and respiration^23^.

### Statistical analysis

The study focused on the evaluation of asthma courses in pregnancy, specifically to identify non-allergic factors that influence its progression. A secondary objective was the influence of asthma on the presence of perinatal complications. Variables included asthma severity according to treatment steps, asthma control according to pACT, presence of exacerbations, presence of asthma worsening during pregnancy, treatment cessation, smoking and environmental tobacco smoke exposure, respiratory tract infections, age over 35 years, and obesity. Variables concerning the presence of perinatal complications included the occurrence of preterm birth, SGA (small for gestational age), miscarriage, gestational diabetes, gestational hypertension, labor induction, assisted labor, cesarean section, and Apgar punctation.

The statistical assessment of confounding factors was not conducted due to the limited number of cases available in the study; however, there was no statistically significant difference between the mentioned cofactors and perinatal complications. Statistical analysis was performed using IBM SPSS 26 program version 26. Qualitive variables are presented as absolute and relative frequencies (%), while quantitative variables are described using χ^2^ test. For continuous variables t-test was applied after verifying normal distribution. To analyze difference between the means of more than two groups (retrospective, prospective, and control) we used one way ANOVA, assuming normal population distribution, equal variances and independent data.

## RESULTS

A total of 83 patients were included in the study, of which 52 patients met the inclusion criteria for the prospective group. However, 12 of the patients did not respond to telephone interviews until the end of pregnancy and, therefore, were moved to the retrospective group. On the other hand, 25 patients were included in the retrospective group according to settings.

The basic characteristics concerning demographic, obstetric data, and asthma course are shown in the Tables 1 and 2. Patients were mostly young, under 35 years (mean=31, SD=5.7, range: 18–38) and predominantly with tertiary education (n=68; 81.9%). They were predominantly multiparas (n=52; 62.0%), with a wide range of gestational weeks at birth (mean=38.57, SD=1.8, range: 34–41) with mild asthma (n=38; 42.2%), and good (n=47; 56.6%) or partially controlled (n=26; 31.3%) asthma.

**Table 1:** Maternal baseline sociodemographic characteristics and history related to pregnancy and birth of the studied group

*Characteristics*	*Total cases (N=83)*	*Prospective cases (N=36)*	*Retrospective cases (N=47)*	*Controls (N=83)*
**Age** (years), mean (SD), range	31 (5.73) 18–38	32 (4.52) 18–38	31 (4.80) 19–37	29 (6.58) 18–40
**Tertiary education**, n (%)	68 (81.90)	27 (75.00)	41 (87.20)	45 (54.20)
**Primiparas** (%)	38.00	30.50	42.50	45.00
**Delivery by cesarean section** (%)	54.20	69.44	52.55	24.10
**Newborn birth weight** (g)	3238.13	3225.56	3680	3317.31
**Gestational age at birth** (weeks), mean (SD), range	38.57 (1.81) 34–41	38.64 (1.91) 35–41	38.48 (1.95) 34–41	38.49 (1.77) 35–41

**Table 2:** Asthma characteristics of the studied group

*Characteristics*	*Prospective cases (N=36)*		*Retrospective cases (N=47)*		*Total cases (N=83)*	
	*n*	*%*	*n*	*%*	*n*	*%*
**Asthma severity**						
Mild	16	44.45	22	46.80	38	45.80
Moderate	16	44.45	19	40.40	35	42.20
Severe	4	11.10	6	12.80	10	12.00
**Change of asthma control during pregnancy**						
Alleviation	3	8.30	20	42.50	23	27.70
Aggravation	15	41.70	7	15.00	22	26.50
No change	18	50.00	20	42.50	38	45.80
**Change of asthma treatment during pregnancy**						
Withdrawal	10	27.00	9	19.14	19	22.80
Intensification	6	16.60	4	8.50	10	12.00
**Adequacy of asthma**						
Inadequately treated	8	22.20	3	6.30	11	13.20

Infections occurred in 23 patients (27.7%), 11 in the perspective (30.6%), and 12 in the retrospective group (25.5%). Infection had been statistically significantly more frequent among patients with partially and uncontrolled asthma (χ^2^=8.504, p<0.05). No statistically significant difference was found between infection episode and perinatal complications.

In 19 cases (22.8%), patients quit their regular treatment after pregnancy was confirmed. We found a statistically significant association between treatment cessation and lower levels of education. The treatment was ceased by 7 patients with tertiary education and 12 patients with secondary and primary education (χ^2^=16.38, p<0.01), data not shown. No statistically significant difference was found between treatment withdrawal nor exacerbation occurrence, reported by 10 patients (χ^2^=1.106, p=0.29).

In 22 cases (26.5%), a worsening of asthma control during pregnancy was reported in the questionnaire and 48 patients (57.8%) experienced at least one exacerbation episode. Overall number of exacerbations was 51 (3 patients experienced exacerbations twice during pregnancy). Most of the exacerbations were mild (40 cases, 78.40% of reported exacerbations), with 20 reported in the prospective (74.1%) and 20 in the retrospective (83.3%) group. Moderate and severe exacerbations occurred in 10 cases, 6 in the prospective (22.20%) and 4 in the retrospective group (16.70%). Severe exacerbation was reported in the prospective group. Among patients with exacerbation episodes, we did not observe a statistically significant increase in perinatal complication prevalence.

None of the pregnant asthmatic patients declared smoking during pregnancy; however, 9 women in the control group (10.84%) declared active smoking. The prevalence of smoking was found to be statistically significantly lower among individuals in the asthmatic group compared to those in the non-asthmatic group (χ^2^=9.52, p<0.01). Secondhand smoke exposure was reported in the questionnaire by 8 women (9.63%) in the cases and by 25 in the controls (30.12%). Five asthmatic patients exposed to SHS (62.5%) declared exacerbation of asthma; however, no statistically significant difference was observed between non-smoking and smoke-exposed patients in terms of asthma control or the presence of exacerbations. Among patients with environmental smoke exposure no perinatal complications were observed in the group of asthmatic patients. Mean Apgar score was 10 (SD=0). In exposed patients in the control group, 2 had induced vaginal deliveries (8.0%), 2 assisted vaginal deliveries (8.0%), 2 miscarriages (8.0%), and one patient (4.0%) suffered from gestational diabetes. The mean Apgar score was 9.72 (SD=0.81). No statistically significant difference was found between SHS and perinatal complications in the studied group. Among smoking controls statistically more frequent occurrence of preterm birth was observed (χ^2^=6.67, p=0.01); however, there was a significant disproportion in compared groups.

Certain complications of pregnancy were observed (Table 3). The most prevalent outcomes in our study were cesarean section (45 cases, 54.20%), preterm birth (4 cases, 4.81%), gestational diabetes (4 cases, 4.81%) and small for gestational age (4 cases, 4.81%). Cesarean section was found to be statistically significantly more frequent among patients with asthma (χ^2^=16.38, p<0.01).

**Table 3:** Pregnancy complications observed in the studied and control group

*Complication*	*Prospective cases (N=36)*		*Retrospective cases (N=47)*		*Total cases (N=83)*		*Total controls (N=83)*		*χ^2^ p (retrospective vs prospective)*	*χ^2^ p (cases vs controls)*
	*n*	*%*	*n*	*%*	*n*	*%*	*n*	*%*		
Preterm birth	2	5.55	2	4.25	4	4.81	4	4.81	0 1	0 1
Small for gestational age	2	5.55	2	4.25	4	4.81	3	3.61	0 1	0.15 0.69
Miscarriage	1	2.77	1	2.12	2	2.40	3	3.61	0 1	0.2 0.64
Gestational diabetes	4	11.11	0	0	4	4.81	4	4.81	5.48 <0.05	0 1
Gestational hypertension	1	2.77	1	2.12	2	2.40	3	3.61	0 1	0.2 0.64
Labor induction	1	2.77	1	2.12	2	2.40	1	1.20	0 1	0.3 0.56
Assisted vaginal delivery	1	2.77	0	0	1	1.20	2	2.40	1.32 0.26	0.3 0.56
Cesarean section	25	69.44	20	52.55	45	54.20	20	24.10	5.94 <0.05	16.37 <0.01
Apgar mean score	9.53		9.93		9.74		9.29		t=2.36* <0.05	t=1.56 0.59

* Significant for retrospective group.

The relative ratio for cesarean section was 2.25. Cesarean section was performed more frequently among patients with severe (χ^2^=7.07, p<0.01) and uncontrolled (χ^2^=6.97, p<0.05) asthma. The Apgar score was statistically significantly worse in patients with moderate and severe asthma (χ^2^=20.37, p<0.05). The prevalence of other perinatal complications was equal in both groups without statistical significance (Table 4).

**Table 4:** Presence of other chronic diseases and perinatal complications in the studied group

*Complications*	*Absence chronic diseases (N=38)*		*Presence of chronic disease (N=45)*		*χ^2^*	*p*
	*n*	*%*	*n*	*%*		
Preterm birth	2	5.30	2	4.40	0.03	0.87
Low birth weight	0	0	4	8.80	3.54	0.06
Gestational diabetes	3	7.90	1	2.20	1.46	0.23
Miscarriage	0	0	2	4.40	1.67	0.19
Gestational hypertension	0	0	2	4.40	1.73	0.19
Inducted delivery	1	2.6	1	2.20	0.02	0.91
Assisted vaginal delivery	0	0	1	2.20	0.86	0.35
Cesarean section	23	60.5	22	48.8	0.13	0.72

No statistically significant differences were found in age, Apgar and perinatal outcomes and complications between the groups, with the exclusion of cesarean section and gestational diabetes (Table 4).

## DISCUSSION

The primary objective of our study was the evaluation of non-allergic factors that may influence the asthma course during pregnancy. The most significant non-allergic factor influencing the course of asthma during pregnancy was occurrence of patient-reported episodes of respiratory infection, reported by almost 30% of patients. The prevalence of infections in previously published studies varies. In a prospective study by Murphy et al.^24^, the overall incidence of questionnaire-reported infections was over 71%, with about one-third of reported infections confirmed by PCR (polymerase chain reactions) swabs for respiratory viral infections. One-third of patients with PCR-confirmed infections reported exacerbation incidence. In our study almost half of patients reporting infections, linked it with exacerbation occurrence (data no shown). In another prospective study by Minerbi-Codish et al.^25^, the incidence of upper respiratory tract and urinary tract infections (defined according to symptomatic criteria) was 36% and was notably higher among pregnant patients with asthma. The differences in outcomes across studies may be attributed to several factors, such as vaccination status, behavioral disparities impacting infection susceptibility, or the timing of pregnancy overlapping with periods of higher infection prevalence. In our study, the overall incidence of infections was relatively low. However, almost half was considered as an exacerbation trigger, suggesting the significance of prioritizing preventive measures, such as vaccinations and the targeted application of face masks, to enhance perinatal outcomes.

Another identified factor was cessation of treatment without consultation with medical personnel, observed in over 20% of cases, and convergent with available studies. Treatment withdrawal has been indicated by numerous studies utilizing medication prescription databases^15,26^. Dutch researchers have indicated that approximately 30% of patients did not fulfill prescriptions for recommended anti-asthmatic treatments following pregnancy diagnosis, suggesting that only to some extent this effect may be attributed to the relief of asthma symptoms during pregnancy. French authors highlight that over 25% of patients did not receive prescriptions for their pre-pregnancy anti-asthmatic treatments, and half of the women were receiving them irregularly. The percentage of patients who discontinued therapy in our study aligns with existing data. The discontinuation of treatment was not associated with previously identified adverse effects, such as the exacerbation of asthma^5^ or perinatal complications^27^. This phenomenon could be explained by the prompt reintroduction of treatment after consultation with an allergist. These findings underscore the necessity education concerning asthma treatment safety of pregnant women with asthma and for healthcare providers.

A secondary objective was to investigate the influence of asthma on the occurrence of perinatal complications. The overall prevalence of perinatal complications among asthmatic patients in our study was low, comparable to that observed in the control group and recent studies. The possible explanation could be predominantly good asthma control, low prevalence of severe exacerbations, and higher level of education among patients in studied group. However, we observed significantly lower Apgar score among group with moderate and severe asthma, what could be a result of insufficient asthma control. Lower Apgar score was also observed in previous studies^11,28^. Other complications included preterm birth, small for gestational age (SGA), and gestational diabetes, each occurring in nearly 5% of cases in our studies, which is comparable to available studies. Bakhireva et al.^10^ reported about 6% preterm birth in subgroup of well controlled asthma. Ali et al.^3^ reported about 6% of preterm birth and 2.6% of gestational diabetes. Yland et al.^29^ reported 4% of SGA. We also observed high prevalence of cesarean sections among asthmatic patients (>50%), whereas other studies have reported a prevalence of around 20%^5^. Although cesarean sections were found to be significantly more prevalent among patients with asthma, these findings need cautious interpretation. The study did not adjust for confounding factors for cesarean section. Furthermore, the data should be considered together with the higher rate of cesarean sections observed in Poland (about 50% in 2023)^30^.

Most of the previous studies have shown elevated rates of smoking among pregnant women with asthma^1^. None of the patients in our study reported smoking, which is consistent with findings by Ali et al.^3^ where only 4.2% of asthmatic patients declared smoking. However, these data rely on patient self-report, and the actual prevalence could be higher. SHS exposure was reported by almost 10% of patients. A relatively high prevalence of smoking (>10%), and SHS exposure (>30%) was noted in the control group. Smoking prevalence is comparable to available European data for pregnant women (3–18%)^31^. These data suggest the important role of smoking and ETS prevention in proper pregnant patient evaluation, not only with concomitant asthma.

Our study suggests that certain factors may play an important role in prevention of perinatal complications, with a strong emphasis on adequate asthma treatment and treatment adherence as a cornerstone. Also, patients’ education concerning asthma treatment, smoking and ETS exposure and infection protective procedures in peri-conceptional period may play a crucial role by improving patients’ awareness.

### Strengths and limitations

The strengths of the study include prospective recruitment of a portion of the samples. This methodology improves the reliability of collected data by reducing recall bias and implementing real-time data collection practices. The study provides preliminary data on subjects not previously explored in the Polish population, concerning asthma in pregnancy, and prevalence of smoking during pregnancy. However, our study exhibits several limitations that warrant consideration. Retrospective recruitment of a portion of the sample recruitment poses a potential limitation due to the risk of selection bias or incomplete data, especially if the documentation is not comprehensive. However, this study mitigated this risk by enrolling participants retrospectively only if complete medical records were available. Moreover, due to the limited number of occurrences in specific variables, adjusting for confounding factors was not feasible in our study.

## CONCLUSIONS

Adequate asthma treatment, respiratory tract infections, and smoke avoidance are the most important modifiable factors that may prevent perinatal complications; however, even mild and well controlled asthma increase the risk of cesarean section.

## Data Availability

The data supporting this research are available from the authors on reasonable request.
